# Insight into the Charge-Ratio-Tuned Solar Vapor Generation of Polyion Complex Hydrogel/Coal Powder Composites

**DOI:** 10.3390/polym15112449

**Published:** 2023-05-25

**Authors:** Zhiteng Ji, Jianhang Zhao, Shanhao Feng, Fengbo Zhu, Wenwen Yu, Yanan Ye, Qiang Zheng

**Affiliations:** 1 College of Materials Science and Engineering, Taiyuan University of Technology, Taiyuan 030024, China; zhitengvip@foxmail.com (Z.J.); zhaojianhang0928@163.com (J.Z.); fengshanhao0147@link.tyut.edu.cn (S.F.); yuwenwen@tyut.edu.cn (W.Y.); yeyanan@tyut.edu.cn (Y.Y.); 2 Shanxi-Zheda Institute of Advanced Materials and Chemical Engineering, Taiyuan 030032, China; 3 Department of Polymer Science & Engineering, Zhejiang University, Hangzhou 310027, China

**Keywords:** polyion complex, charge ratio, solar vapor generation, water purification

## Abstract

Solar-driven water purification has been deemed a promising technology to address the issue of clean water scarcity. However, traditional solar distillers often suffer from low evaporation rates under natural sunlight irradiation, while the high costs of the fabrication of photothermal materials further hinders their practical applications. Here, through the harnessing of the complexation process of oppositely charged polyelectrolyte solutions, a polyion complex hydrogel/coal powder composite (HCC)-based highly efficient solar distiller is reported. In particular, the influence of the charge ratio of polyanion-to-polycation on the solar vapor generation performance of HCC has been systematically investigated. Together with a scanning electron microscope (SEM) and the Raman spectrum method, it is found that a deviation from the charge balance point not only alters the microporous structure of HCC and weakens its water transporting capabilities, but also leads to a decreased content of activated water molecules and enlarges the energy barrier of water evaporation. As a result, HCC prepared at the charge balance point exhibits the highest evaporation rate of 3.12 kg m^−2^ h^−1^ under one sun irradiation, with a solar–vapor conversion efficiency as high as 88.83%. HCC also exhibits remarkable solar vapor generation (SVG) performance for the purification of various water bodies. In simulated seawater (3.5 wt% NaCl solutions), the evaporation rate can be as high as 3.22 kg m^−2^ h^−1^. In acid and alkaline solutions, HCCs are capable of maintaining high evaporation rates of 2.98 and 2.85 kg m^−2^ h^−1^, respectively. It is anticipated that this study may provide insights for the design of low-cost next-generation solar evaporators, and broaden the practical applications of SVG for seawater desalination and industrial wastewater purification.

## 1. Introduction

With the rapid development of modern industry and the continuous growth of the population [1], fresh water scarcity has been more severe than ever [2]. Solar-driven interfacial evaporation for efficient seawater desalination/wastewater purification has been considered a reliable technology to alleviate the water shortage issue, because it is widely available, sustainable, and ecofriendly [3]. In the past decade, people have developed various materials with high solar–thermal conversion efficiency. For example, carbon materials are the most competitive candidates due to their excellent photothermal conversion ability and versatile processabilities [3,4]. Sun et al. reported on a flexible graphene oxide nanoribbon paper (GONRs paper), which could realize continuous clean water production from seawater and simulated wastewater with nearly 91.5% photothermal conversion efficiency under one sun irradiation (1 kW m^−2^) [5]. Lu et al. fabricated a MXene/rGO hybrid solar vapor generation (SVG) device with concave pyramid-shaped surface patterns; the obtained evaporator showed an extremely high evaporation rate up to 3.62 kg m^−2^ h^−1^ [6]. Biomass materials [7], in particular woods, hold the advantages of water transporting ability, low thermal conductivity, low density, and remarkable hydrophilicity [3,8]. Hu et al. reported on a high-performance SVG composed entirely of natural wood with an evaporation rate as high as 11 kg m^−2^ h^−1^ under ten sun irradiation (10 kW m^−2^) [9]. Some metal nanoparticles that exhibit unique plasmonic photothermal effects have also been harnessed for efficient solar vapor generation, such as the 3D self-assembly of aluminium nanoparticles [10] and flexible thin-film black gold membranes [11]. Wang et al. observed that Ti_2_O_3_ nanoparticles of outstanding photothermal conversion ability can be used for efficient SVG [12]. Despite the tremendous endeavors in improving the photothermal conversion ability of SVG, the low evaporation rate (<1.6 kg m^−2^ h^−1^ under 1 sun), as well as the high cost of the fabrication process, greatly hinder their practical applications.

Hydrogels, which are composed of cross-linked hydrophilic polymer networks with a large amount of entrapped water [13], have been demonstrated as new materials for highly efficient solar-driven water purification [14]. Compared with traditional SVG materials, hydrogels have widely adjustable physical and chemical properties [15,16], which endow them with many additional merits such outstanding processability [17] and self-healing ability [18]. For instance, by utilizing 3D printing technology, hydrogels can be processed into desired shapes for enhanced light absorption and accelerated water transporting properties to meet the demands of SVG [19]. More importantly, the tunable interactions between the hydrophilic polymer skeleton and the surrounding water molecules can reduce the energy required for water evaporation [20,21]. Therefore, through the rational design of hydrogel materials and the optimization of thermal management [22], hydrogel-based SVG can break through the evaporation rate limit of traditional photothermal materials. For example, Yu et al. [23] reported a hierarchically nanostructured gel (HNG) based on poly(vinyl alcohol) (PVA) and poly(pyrrole). Since the activation of surrounding water molecules by PVA networks greatly reduces the evaporation enthalpy of water in HNG, the evaporation rate of the device can be as high as 3.2 kg m^−2^ h^−1^ under one sun illumination. The authors further designed hydrogel evaporators with hydrophobic island-shaped patches that achieved a record high evaporation rate of 4.0 kg m^−2^ h^−1^ with 93% efficiency under one sun irradiation [24]. Recently, polyion complex (PIC) hydrogel-based SVG has gained increasing attentions for solar desalination due to its significantly improved SVG performance in brines. The unique salt responsive characters of PIC could alter the water state, resulting in the enhanced hydration property of the polymeric skeleton in brine. As a result, PIC-based SVG could achieve an evaporation rate of 2.79 kg m^−2^ h^−1^ in simulated seawater (3.5 wt% NaCl solutions) under one sun illumination, which was 67% higher than that in pure water [25]. Although hydrogel-based SVG has gained increasing interest, it has been a challenge to construct highly efficient SVG with low-cost photothermal materials. Meanwhile, it is essential to clarify the correlations between the compositions and the water state variations inside hydrogel-based SVG, as well as the subsequent solar-driven water purification performance.

In this paper, by incorporating PIC as a gel matrix, with low-cost coal powders as efficient solar absorbents, a hydrogel/coal powder composites (HCC)-based SVG has been prepared for efficient solar driven water purification. In particular, we investigated the effect of the charge ratio of polyanions-to-polycations on the SVG performance of HCC. The results show that when the charge ratio of polyanions-to-polycations is near the charge balance point, the average evaporation rate of HCC can reach a maximum of 3.12 kg m^−2^ h^−1^ at one sun irradiation, and the energy conversion efficiency can be up to 88.83%. By investigating the variations of water states and water transporting rates in HCC networks, the mechanism for the charge-ratio-dependent SVG performance was explored. Furthermore, we investigated the purification performance of HCC in various water bodies. In simulated seawater (3.5 wt% NaCl solutions), the evaporation rate of HCC can be increased to 3.22 kg m^−2^ h^−1^. In acidic and alkaline solutions, HCC can still maintain a stable evaporation rate of 2.98 and 2.95 kg m^−2^ h^−1^, respectively, and the pH value of the collected purified water is stabilized at about 7, demonstrating the potentials of HCC for seawater desalination and wastewater purification.

## 2. Materials and Methods

### 2.1. Materials

The sodium *p*-styrene sulfonate (NaSS; anionic monomer, 90 wt% purity), methacryloyl propyl trimethyl ammonium chloride (MPTC; cationic monomer, 50% aqueous solution), and α-ketoglutaric acid (99% wt% purity) were purchased from Aladdin (Shanghai, China), the coal was purchased from Shanxi Jincheng Anthracite Mining Group Co., Ltd. (Jincheng, China), NaOH was purchased from Tianjin Kemiou Chemical Reagent Co., Ltd. (Tianjin, China), hydrochloric acid was purchased from Aladdin Chemical Reagent Co., Ltd. (Shanghai, China), and Millipore (Burlington, MA, USA) deionized water was used for all experiments.

### 2.2. Sample Preparation

Synthesis of the polyanions (PNaSS) and polycations (PMPTC): PNaSS and PMPTC were first synthesized by polymerizing the precursor solutions of NaSS and MPTC, respectively. Typically, NaSS (85.68 g), α-ketoglutaric acid (0.027 g), and deionized water (374 mL) were mixed together by sonication (solution A). MPTC (154.62 g), α-ketoglutaric acid (0.027 g), and deionized water (374 mL) were mixed together by sonication (solution B). Then solution A and B were synthesized under UV light irradiation (365 nm wavelength, 7.5 ×10^−3^ W cm^−2^) for 8 h, respectively. Both of the resultant viscous liquids A and B were precipitated in ethanol for 2 h and then dried at 100 °C in a vacuum oven for 4 h to obtain PNaSS and PMPTC powders.

**Figure 1:**
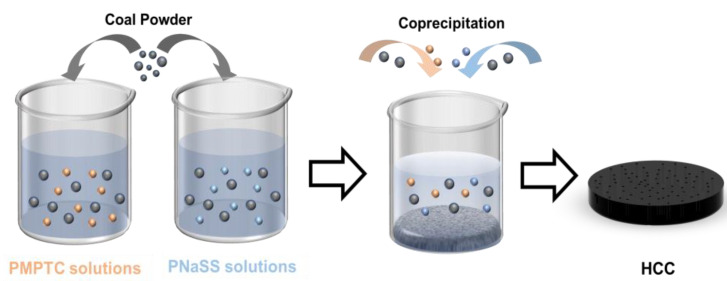
Schematics for the fabrication process of HCC.

Fabrication of HCC: To prepare the HCC (as shown in Figure 1), a certain amount of coal (10 g) particles were put into a planetary ball mill (12LF-P12L, Hunan Focuses Experimental Instrument Co., Ltd., Hunan, China) and ground (with a rotating speed of 500 rpm) for 5 h to produce uniform coal powders (CPs). Next, PNaSS and PMPTC were dissolved separately in deionized water (90 mL) and magnetically stirred for 2 h at a speed of 500 rpm. Then, CPs (0.9 g) was added into PNaSS and PMPTC solutions, respectively. The mixtures were stirred vigorously for 1 h at a predetermined speed (1000 rpm) to ensure complete dispersion of CPs in the polymer solutions. Finally, the mixed solution of CPs and polyelectrolyte was slowly dropped into deionized water (50 mL) under stirring (500 rpm) for 30 min. Afterward, the mixture was left for 8 h for the self-assembly of HCC. The molar concentration ratio of polyanion (PNaSS)-to-polycation (PMPTC) was controlled at 1:1, 1.05:1, 1.1:1, 1.15:1, and 1.2:1 to prepare HCC of different charge ratios. The resulting HCC was dialyzed in deionized water for 3 days, with the water being changed every day. The samples were labeled as HCC-x, where x represented the molar ratio of PNaSS-to-PMPTC. 

### 2.3. Analysis and Characterization

The micro structure of the gels was characterized by using a scanning electron microscope (SEM, Zeiss Gemini 300, Jena, Germany). The samples were prepared by freeze-drying. Before SEM characterization, the surface was coated with a thin layer of gold by sputtering method.

The infrared spectrum of solid PNaSS, PMPTC, and HCC was performed by using a Fourier transform infrared spectrometer (FTIR, BRUKER, Bremen, Germany), the samples were prepared using the potassium bromide pellet method. The scanning range of wave number was 500–4000 cm^−1^.

To determine the saturated water content ($Q_{s}$) and water transporting rate (*V*) of HCC, freeze-dried hydrogel samples were placed into deionized water and allowed to swell. Samples were removed and weighed every minute. This process was repeated until the gels reached swelling equilibrium. $Q_{s}$ was calculated using Formula (1) [26]:

(1) $$Q_{s}=\frac{m_{t}-m_{0}}{m_{0}}$$

where *m*_t_ and *m*_0_ represent the mass of hydrogel in swollen and freeze-dried states at a certain time, respectively. The test was repeated for 3 times.

The water transport capability *V* could then be evaluated by testing the time from a half-swollen state to a fully swollen sate of HCC by Formula (2) [26]:

(2) $$V=\frac{0.5Q_{s}}{t_{h}}$$

where $Q_{s}$ is the saturated water content obtained from Formula (1) and *t*_h_ is the swelling time.

The Raman spectrum of water was obtained using a spectrometer (LabRAM HR Evolution, Horiba Scientific, Zhubei City, Taiwan), with a scanning range from 2800 to 4000 cm^−1^. Similarly, the infrared spectrum of water in HCC was measured using a Fourier infrared spectrometer (FTIR, Bruker Company, Bremen, Germany), with a wavelength scanning range of 2800–3800 cm^−1^. The pH of acid–base solutions and purified solution were determined using a pH meter (METTLER TOLEDO, Shanghai, China).

### 2.4. Solar Vapor Generation Experiment

The solar vapor generation experiment was performed by using a 300 W xenon lamp (CEL-S500/350, CEAULIGHT, Beijing, China) as simulated sunlight. The light intensity was adjusted by tuning the distance between light source and material surface to ensure the output of 1 kW m^−2^ (one standard sunlight) irradiation. The light intensity was measured with a solar power meter (CEL-FZ-A). HCC of 3 mm thickness was cut into rectangular pieces with an area of 4 cm × 1.5 cm, and each piece was put on top of a PS foam of the same size. Mass loss caused by water evaporation under 1 kW m^−2^ sunlight was measured using an electronic balance (AL204, METTLER TOLEDO, Shanghai, China) at 10-min intervals. The environment temperature was maintained at 20 °C, and relative humidity was ~60%. All evaporation rates were measured after a stabilization period of 30 min. The solar–vapor conversion efficiency (*η*) was calculated using Formula (3) [27]:

(3) $$\eta =\frac{\dot{m}H_{e}}{P_{0}}$$

where $\dot{m}$ is the mass flux (after subtracting the mass flux of water evaporation under dark environment), *H*_e_ is the equivalent evaporation enthalpy of HCC in pure water, which was calculated as shown in Formula (4) below [18], and *P*_0_ is the solar radiation power (1 kW m^−2^).

(4) $$U_{\mathrm{in}}=H_{v}m_{0}=H_{e}m_{g}$$

where $U_{\mathrm{in}}$ is the identical power input, *H*_v_ and *m*_0_ are the evaporation enthalpy and mass change in bulk water, and *m*_g_ is the mass change in HCC. 

## 3. Results

**Figure 2:**
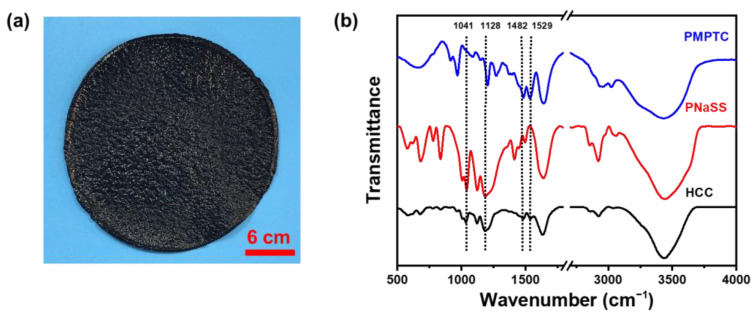
(**a**) Photograph of a typical HCC sample. (**b**) FTIR spectra of dried PMPTC, PNaSS, and HCC.

To enable the functionalization of PIC with photothermal conversion abilities, home-made CPs were evenly dispersed into PNaSS and PMPTC solutions. After which, the obtained PNaSS/CPs and PMPTC/CPs solutions were mixed spontaneously to enable the complexation between PNaSS and PMPTC, which produced black HCC (Figure 2a). The formation of intermolecular ionic bonds was characterized by FTIR. As shown in Figure 2b, the peaks at wave numbers of 1482 cm^−1^ and 1529 cm^−1^ correspond to the bending of trimethylamino (-N^+^(CH_3_)) of PMPTC and the stretching of amido CON-H, respectively [28]. The peaks at 1041 cm^−1^ and 1128 cm^−1^ correspond to the symmetric and asymmetric stretching of the sulfonic acid group of PNaSS, respectively [29,30]. In HCC gel, the peak of the sulfonic acid group shifted towards 1035 cm^−1^ and 1123 cm^−1^, indicating the successful formation of ionic bonds between PNaSS and PMPTC.

**Figure 3:**
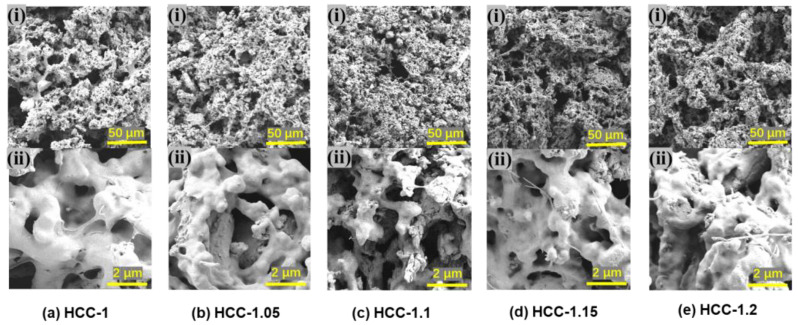
SEM photos of HCC: (**a**) HCC-1, (**b**) HCC-1.05, (**c**) HCC-1.1, (**d**) HCC-1.15, and (**e**) HCC-1.2.

Since the formation of HCC is based on the complexation of PNaSS and PMPTC due to electrostatic interactions, the charge ratio of polyanions-to-polycations could affect the microstructures, as well as the SVG performance, of HCC. The morphology of hydrogels with varied charge ratios was first observed through SEM. As shown in Figure 3, HCC obtained from different charge ratios all exhibited porous structures. CPs distributed uniformly in the gel matrix, which should be helpful to ensure photothermal conversion abilities. However, HCC-1.1 showed most prominent micropores, and a deviation from this charge ratio lead to decreased porosity. According to our previous research [31], the charge balance point of this PIC hydrogel appears when the molar ratio of PNaSS-to-PMPTC is 1.1:1. Deviation from this point would cause Coulomb repulsion due to the excess level of ionic groups, leading to a slight expansion of the HCC and decreasing of their porosity.

**Figure 4:**
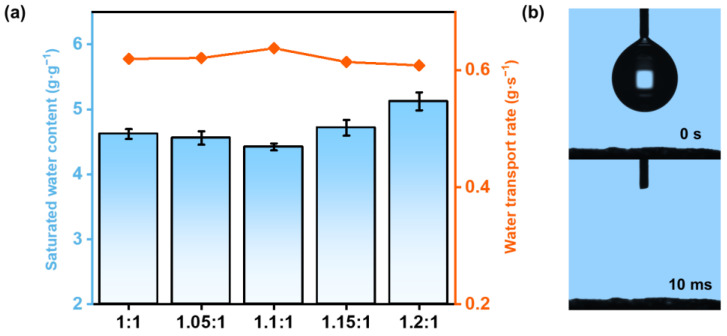
(**a**) The swelling ratio and water transport rates of HCC in pure water. (**b**) Hydrophilicity of HCC through a water contact angle test.

This varied porous structure induced by different charge ratios may alter the water absorbing ability, as well as the water transporting ability of HCC [32], which is crucial for solar-driven water evaporation. Figure 4a presents the statistical results of the saturated water content of HCC (analyzed through an ordinary one-way ANOVA, with Tukey’s correction for multiple comparisons between groups, the values are mean ± SD, *n* = 4, *p* < 0.05). Statistical differences were found for the saturated water content, indicating a charge-ratio-dependent water absorbing ability. As the charge ratio increased, the saturated water content exhibited a minimum at HCC-1.1 (4.42 ± 0.05 g g^−1^). This should be due to the fact that excess levels of either ionic polymer will bring extra water.

To evaluate the water transporting capability of HCC, we recorded its swelling time from a half-swollen state to a fully swollen state, which showed the opposite trend to the saturated water content (Figure 4a). With increasing charge ratio, the water transport rate came to a maximum at HCC-1.1 (0.64 ± 0.02 g s^−1^). Together with the SEM results, it could be concluded that, the HCC-1.1 with the maximum porosity enables the fastest water transporting, which should be beneficial for continuous SVG. The excellent water transporting performance was further verified through a water contact angle test (Figure 4b), where a water droplet was instantly absorbed by HCC-1.1 within only 10 ms.

**Figure 5:**
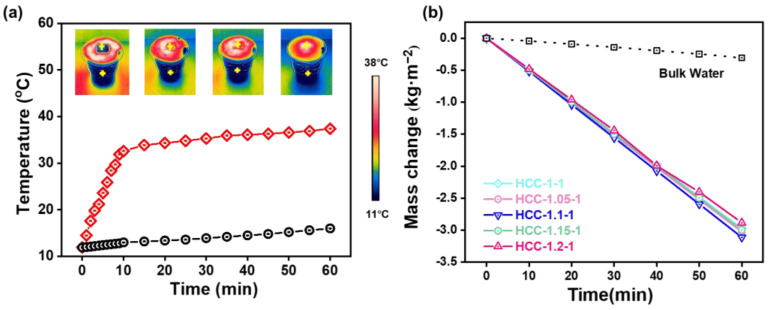
(**a**) Temperature evolutions of the HCC surface and bulk water. Insets show the infrared images of the temperature distributions at different time points. (**b**) Mass change in evaporated vapor of HCC in pure water over time.

The SVG performance of HCC was then evaluated under one sun illumination. Before being subject to simulated sunlight, a piece of HCC was placed on a PS foam to enable localized thermal heating to promote energy utilization efficiency. After which, the temperature evolution of bulk water and the surface of HCC were monitored using an infrared thermal imager. The infrared photos in Figure 5a demonstrated that HCC has good photothermal conversion ability. With continuous light illumination, the surface temperature of HCC rapidly increased from 11.9 °C to 32.6 °C within 10 min, and then gradually stabilized to 37.4 °C within 60 min, whereas the temperature of bulk water only slightly increased to 16 °C within 60 min. Based on this design, we explored the influence of charge ratios on the SVG performance of HCC in pure water. As shown in Figure 5b, HCC-based SVG exhibited remarkable evaporation performance under one sun; the evaporation rates of HCC-1, HCC-1.05, HCC-1.1, HCC-1.15, and HCC-1.2 were 2.97, 3.02, 3.12, 2.99, and 2.88 kg m^−2^ h^−1^, respectively, which were far beyond that of pure water (0.31 kg m^−2^ h^−1^) and suggested their great potentials for wastewater purification. Evidently, the above results also indicate that charge ratio could remarkably affect the SVG performance of HCC, which should be attributed to the altered microstructures’ different water transporting abilities, as well as their different interactions with surrounding water molecules [20,23].

**Figure 6:**
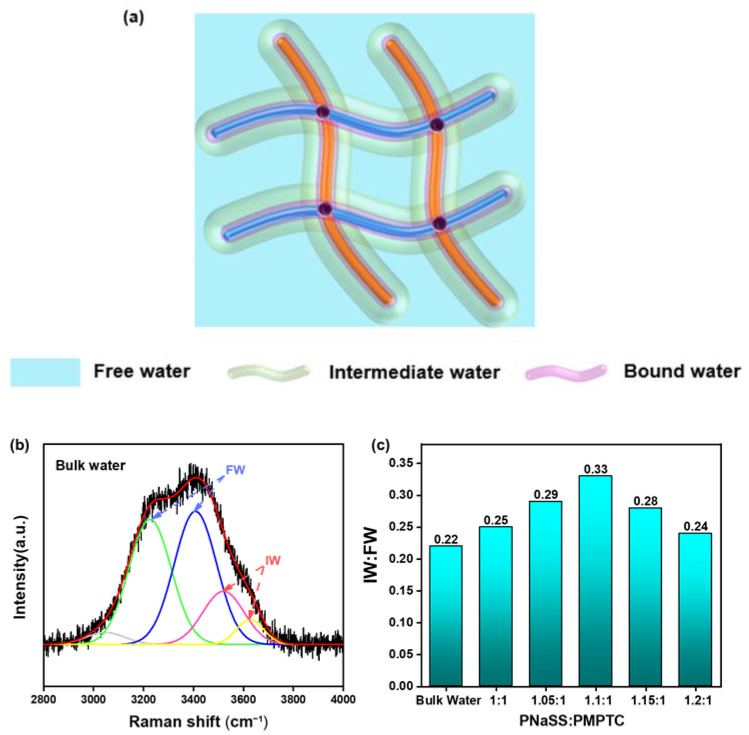
(**a**) Schematic of the water in the hydratable polymer network of the HCC. (**b**) Raman spectra in the energy region of O–H stretching with fitting curves for bulk water. (**c**) The ratio of IW to FW of HCC with different charge ratios in pure water.

Yu et al. [23] pointed out that water molecules exist in three states in hydrogel networks: free water (FW), bound water (BW), and intermediate water (IW). Since BW is compactly bounded to the hydrophilic polymer chains, they can hardly escape from hydrogels. In comparison, IW is unstable and can escape from the polymer networks with less energy and is more active within hydrogels [20,23]. In terms of HCC, the charge-ratio-dependent SVG performance should also be related to the varied water states inside the gels, as depicted in Figure 6a. To track the underlying mechanism of the varied evaporation rates of different HCC, we analyzed the O-H stretching vibration spectrum of water molecules with a Raman spectrometer and extracted the relative amount of IW to FW in HCC [33,34]. Typical Raman spectrum of pure water is shown in Figure 6b. The peaks at 3210 and 3410 cm^−1^ represent free water (FW) with four hydrogen bonds, two protons, and two electron pairs with adjacent water molecules. The peaks at 3527 and 3610 cm^−1^ correspond to the intermediate water (IW) that forms weak hydrogen bonds with surrounding water molecules [16,28]. We obtained the IW:FW ratio in HCC under different charge ratios through similar methods and illustrated it in Figure 6c. In bulk water, the IW:FW ratio was only 0.22, which is consistent with previous reports [23,25]. However, the IW:FW ratio of HCC increased first and then decreased with an increase in charge ratio, indicating that the charge ratio does affect the states of water in HCC. The IW:FW ratio reached a maximum of 0.33 when the charge ratio came to the charge balance point (HCC-1.1).

**Figure 7:**
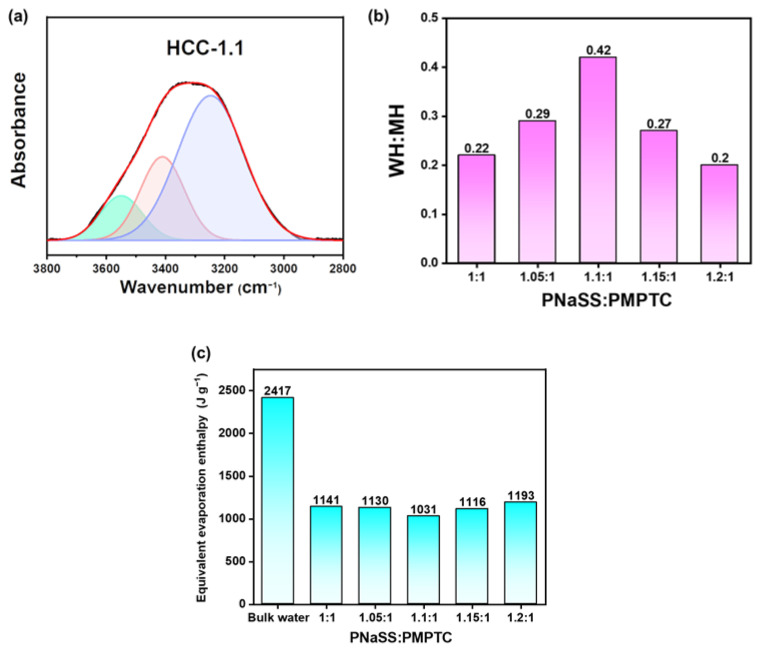
(**a**) The fitted FTIR spectra of HCC in the range between 3800 and 2800 cm^−1^ (blue: SH, red: MH, green: WH) (**b**) The ratio of WH-to-MH of HCC with different charge ratios in pure water. (**c**) The equivalent water vaporization enthalpy of bulk water and pure water in HCC.

The charge-ratio-altered water states in HCC were further investigated using the FTIR method in the range of 3800–2800 cm^−1^. It has been documented that, in terms of pure water, there would appear two peaks for the hydrogen bonds (HB) at 3230 (strong HB, SH) and 3410 cm^−1^ (medium HB, MH) [35]. Figure 7a illustrates the typical FTIR spectrum of HCC-1.1; notably, a new peak at around 3583 cm^−1^ (weak HB, WH) appeared, as the bands in the range between 3800 and 2800 cm^−1^ could be well fitted with three Gaussian curves. The WH:MH ratio in HCC was further calculated and shown in Figure 7b; as the charge ratio came to 1.1:1 (charge balance point), the WH:MH came to a maximum (0.42), and a deviation from the charge balance point led to a decreased amount of activated water molecules. This trend is greatly consistent with the Raman results, as MH and WH correspond to the FW and IW inside HCC, respectively. The charge-ratio-altered water states in HCC could lead to varied energy being required for water evaporation, and thus improving its SVG performance. The equivalent evaporation enthalpy [36] of pure water in different HCC samples was then measured. As shown in Figure 7c, compared with bulk water, all the HCC samples exhibited remarkably lowered equivalent evaporation enthalpy of water, which reached a minimum of 1031 J g^−1^ for HCC-1.1, consistent with the Raman and FTIR results.

**Figure 8:**
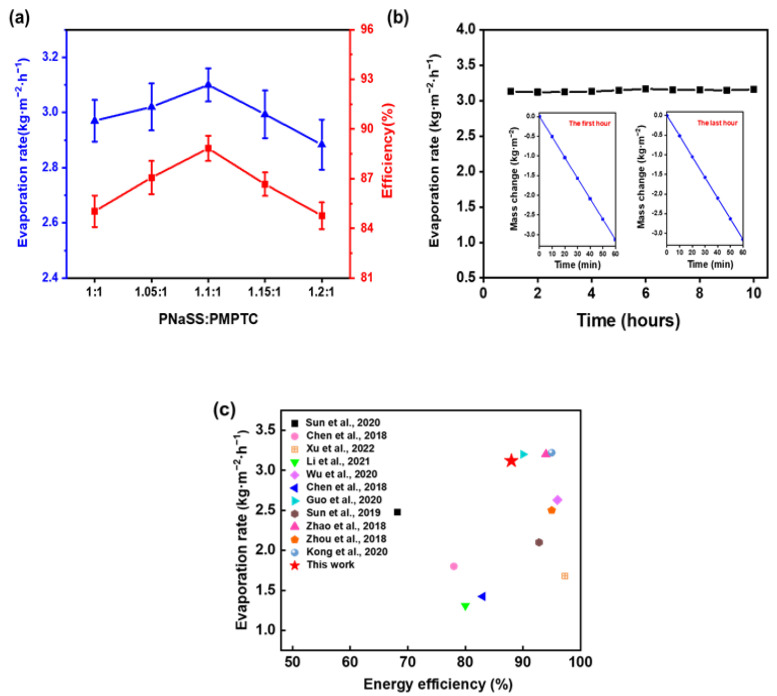
(**a**) The water evaporation rate and energy efficiency of HCC of different charge ratios in pure water. (**b**) Evaporation rates of HCC in pure water within 10 h (insets show the mass change in water in the first hour and the last hour, respectively). (**c**) Comparison of HCC SVG performance and previous reports under one sun (Sun et al., 2020 [37]; Chen et al., 2018 [38]; Xu et al., 2022 [18]; Li et al., 2021 [39]; Wu et al., 2020 [40]; Chen et al., 2018 [41]; Guo et al., 2020 [42]; Sun et al., 2019 [36]; Zhao et al., 2018 [23]; Zhou et al., 2018 [43]; Kong et al., 2020 [44]).

The energy conversion efficiency is an important parameter to access the SVG performance of HCC. As illustrated in Figure 8a, HCC-1.1 showed the fastest evaporation rate with a highest efficiency of 88.83%, consistent with the trend of the charge-ratio-dependent SVG. In the following, HCC-1.1 was selected for further SVG tests. To evaluate the stability of the SVG performance of HCC, a continuous 10 h evaporation test was performed. As shown in Figure 8b, the evaporation rate maintained stable in the range of 3.1 ± 0.1 kg m^−2^ h^−1^, indicating that HCC-1.1 has excellent evaporation stability and durability. Furthermore, the performance of the developed HCC was compared with previously reported materials in Figure 8c; HCC-based SVG achieved a competitively high evaporation rate and high energy utilization efficiency.

**Figure 9:**
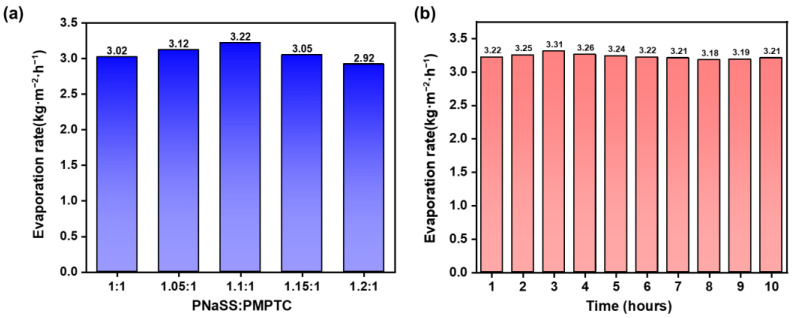
(**a**) The evaporation rate of HCC with different charge ratios in 3.5 wt% NaCl solution. (**b**) Evaporation rates of HCC-1.1 in 3.5 wt% NaCl solution within 10 h.

Next, the purification ability of HCC on simulated seawater was accessed. As shown in Figure 9a, the evaporation rate of HCC in simulated seawater (3.5 wt% NaCl solution) was slightly higher than that in pure water and, regardless of the charge ratio, a maximum evaporation rate of 3.22 kg m^−2^ h^−1^ appeared for HCC-1.1. This could be attributed to the unique interactions between the charged groups of PIC and salt ions and water molecules, which led to the improved hydration properties of the polymer networks, effectively activating the water molecules and reducing the energy required for evaporation [18]. Additionally, a continuous desalination test was performed on HCC-1.1. As shown in Figure 9b, the evaporation rate remained unchanged during the test, with no salt deposition on the surface, suggesting its reliability for seawater desalination.

**Figure 10:**
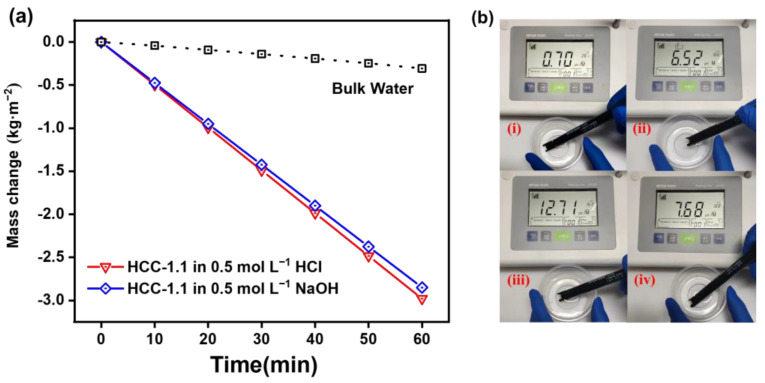
(**a**) Mass change in HCC-1.1 in 0.5 M HCl and NaOH (light intensity: 1 kW m^−2^). (**b**) pH value of the simulated wastewater of HCl (**i**) and NaOH (**iii**), and resultant purified water (**ii**) and (**iv**).

The evaporation rates of HCC-1.1 in acid/alkaline aqueous solutions were also tested to evaluate its purification ability for simulated industrial wastewater. Before and after purification, the pH value, which is an indicator for the quality of water body, was recorded. As shown in Figure 10a, under one sun irradiation, the evaporation rate of HCC-1.1 in 0.5 mol L^−1^ hydrochloric acid and 0.5 mol L^−1^ sodium hydroxide was 2.98 and 2.85 kg m^−2^ h^−1^, respectively, which is slightly lower than that in pure water. Additionally, after purification, the collected purified water exhibited a stale pH value of ~7. These results (Figure 10b) demonstrate the good capability for HCC to treat harsh water bodies, indicating their great potential for industrial wastewater purification.

## 4. Conclusions

In summary, an HCC-based highly efficient SVG, composed of PIC skeletons and low-cost CPs as light absorbents, has been reported. In particular, the influence of charge ratio on the SVG performance has been well studied. Alternating the charge ratio of the oppositely charged polyelectrolyte led to the formation of HCC of varied microstructures, as well as different water states inside the gels. HCC fabricated from the charge balance point exhibited the most prominent water-transporting abilities, while the water molecules inside the gel showed the most activated state, therefore greatly improving the SVG performance of HCC. As a result, HCC obtained from the charge balance point showed the highest evaporation rate of 3.12 kg m^−2^ h^−1^, and the highest energy conversion efficiency—up to 88.83% under one sun illumination. Deviation from the charge balance point led to a weakened SVG performance. HCC also demonstrated a high and stable evaporation rate in simulated seawater and wastewater bodies. This concept of HCC may not only bring insights into the design of low-cost next-generation SVG, but would also broaden the practical applications of SVG for seawater desalination and industrial wastewater purification.

## Data Availability

The data that support the findings of this work are available from the corresponding author on reasonable request.
